# Prompt letters to reduce non-attendance: applying evidence based practice

**DOI:** 10.1186/1471-244X-8-90

**Published:** 2008-11-16

**Authors:** Mahesh Jayaram, Ranganath D Rattehalli, Ihsan Kader

**Affiliations:** 1 Consultant Psychiatrist, Leeds Partnerships NHS Foundation Trust, Bridge House, Balm Road, Leeds LS10 2TP, UK; 2 Specialist Registrar, Leeds Partnerships NHS Foundation Trust, Millfield House, Kirk Lane, Leeds LS19 7LX, UK; 3 Consultant Psychiatrist, Leeds Partnerships NHS Foundation Trust, Bridge House, Balm Road, Leeds LS10 2TP, UK

## Abstract

**Background:**

Non-attendance rates in psychiatric outpatient clinics have been a topic of considerable interest. It is measured as an indicator of quality of service provision. Failed attendances add to the cost of care as well as having an adverse impact on patients leading to missing medications, delay in identifying relapses and increasing waiting list time. Recent trials have demonstrated that prompting letters sent to patients led to a decrease in non-attendance rates. We applied this evidence based practice in our community mental health setting to evaluate its impact.

**Methods:**

Using a before and after study design, we sent prompting letters to all patients due to attend outpatient clinic appointments for a period of six months in 2007. Non-attendance rates were compared with the corresponding period in 2006. We also looked at trends of non-attendance prior to this intervention and compared results with other parts of our service where this intervention had not been applied.

**Results:**

1433 prompting letters were sent out to all out-patient appointments made from June to November 2007. This resulted in an average non-attendance rate of 17% which was significantly less compared to 27% between June and November 2006 (RR 0.65, 95% CI 0.56 to 0.76, NNT 11). No downward trend in non-attendance rate was identified either prior to the intervention or when compared with similar teams across the city.

**Conclusion:**

Prompt letters have been shown to reduce non-attendance rates in previous RCTs and systematic reviews. Our findings demonstrate a reduction in non-attendance rates with prompting letters even under non-trial conditions. Majority of the patients were constant during the two periods compared although there were some changes in medical personnel. This makes it difficult to attribute all the change, solely to the intervention alone. Perhaps our work shows that the results of pragmatic randomised trials are easily applicable and produce similar results in non-randomised settings. We found that prompting letters are a useful and easy to apply evidence based intervention to reduce non-attendance rates with a potential to achieve significant cost savings.

## Background

Outpatient clinic visits are an important point of contact between healthcare professionals and recipients of care. Attendance rates across specialties in the UK have been looked at closely as a measure of quality of care, to identify bottlenecks in the referral pathway between primary and secondary care and to reduce waiting list times [1]. Research in other medical specialities has shown that non-attendance is unrelated to the seriousness of the illness [2] and patients who do not attend may have treatable morbidity [3]. Some patients may make a conscious decision to miss appointments, balancing their decision on the perceived benefits and costs [4]. The commonest reason for non-attendance is forgetting about the appointment and this is particularly linked to non-adherence with medications [5,6]. In mental health, the duration of new appointments is usually longer than follow up appointments [7] and this makes non-attendance for new appointments an even greater waste of already stretched resources and delaying contact with services [6]. For patients with a severe mental illness who are more likely to miss appointments [8], missed appointments equate to missing medications or delays in identifying early warning signs of a relapse and disengagement from services [9].

Combined new and follow-up appointment non-attendance rate is around 12% nationally across all specialities [10]. The cost per lost NHS appointment during 1984 ranged from £20 to £50 [11] which had risen to £65 in 1997 [12] with an estimated total cost of around £300 to £360 million annually [10,12] and this figure may have increased recently. Failed appointments cost each NHS Trust around £1 million per year [13]. More recently, with the emphasis shifting towards reference costs, it is difficult to estimate costs across the country however, in Leeds we estimate that on average a follow up psychiatric outpatient appointment costs around £70 to £80. The Health Care Commission which rates the performance of Trusts report that high non-attendance rates lead to patients missing out on care and those services need to be patient centred. Non-attendance rates are banded on a scale of 1 to 5 (higher the banding = better the performance) and a non-attendance target of ≤ 11.3% is considered acceptable [14].

A systematic review in this area showed that a simple orientation-type letter, sent 24 hours before clinic appointment may encourage attendance [15]. Pooled data from the recent Leeds PROMPTS trial and the existing systematic review demonstrated that prompting letters significantly reduced the non-attendance rates (5RCTs, N = 1184, RR 0.72 95% CI 0.59 to 0.89, NNT 6, CI 4 to 14) and that it was possible to apply this intervention in a busy clinical setting with the use of minimal additional resources [16]. We decided to implement this evidence based intervention of sending prompt letters in our outpatient clinics to see if it would make a difference to non-attendance rates in a pragmatic real world, non-randomised setting.

## Methods

There are two community mental health teams based at Bridge House, South Leeds working in General Psychiatry catering to age groups between 18 and 65, comprising of a mixture of socio economic states, but predominantly economically deprived background [17] and covering a population of about 64,000. Both these teams decided to implement the use of prompt letters. We designed a short letter (see appendix 1) reminding patients of the appointment which took around 30 seconds to read. This was printed on headed paper, explained the time of appointment, and gave the name of doctor, short description of the clinic and its routine, a map and finally a request to bring medication and a friend or family member. The letter was designed in line with the one used in the Leeds PROMPTS randomised study [16]. This letter was individualised to the patient and sent out by the team secretary a week before the scheduled appointment by Royal Mail First Class Post. It is standard practice within our organisation to send out patient appointment letters by post and sending of reminder letters was approved by our organisation. As we were evaluating the effects of a practice that was being implemented, we did not require an ethics committee approval or informed consent. All our letters complied with the Data Protection Act 1998 [18].

The prompt letters began to be rolled out in the last week of May 2007 so that patients due for their appointments from the first week of June 2007 onwards received these letters. This is an ongoing practice now. We compared the non-attendance or DNA (Did Not Attend) rates before and after the implementation of this intervention. If a patient failed to attend the outpatient clinic and no message had been received that this person was not going to attend, the patient was deemed to be a non-attendee or 'DNA'. We compared non-attendance (DNA) rates in the year 2007 against the same months for year 2006 to eliminate any possible seasonal variations in non-attendance rates. We also looked at non-attendance trends across the whole year as well as compared it with another part of Leeds with a similar community mental health team set up and population demographics [19] to see if there was a general tendency of decrease or increase in non-attendance rates. E-care, an outpatient clinic booking tool used by our Trust enabled us to extract the required data.

### Setting

In the context of NHS, most patients are managed in primary care and more complex cases get referred to secondary and tertiary care. Our Community mental health teams are part of secondary care services. All new referrals to our teams go through an initial 'gate keeping' multidisciplinary assessment done by a member of community mental health team and sometimes a medical doctor. Following these assessments, some patients are signposted to other appropriate services and the ones that need more in depth medical assessments are booked into regular outpatient clinics. These clinics constituted our patient sample. The treating teams comprised of junior doctors, educational staff grades and consultants amongst other disciplines. Junior trainees and staff grades change posts once every 6 to 12 months depending on their training requirements and there was one change of consultant in March 2007.

## Results

**Figure 1:**
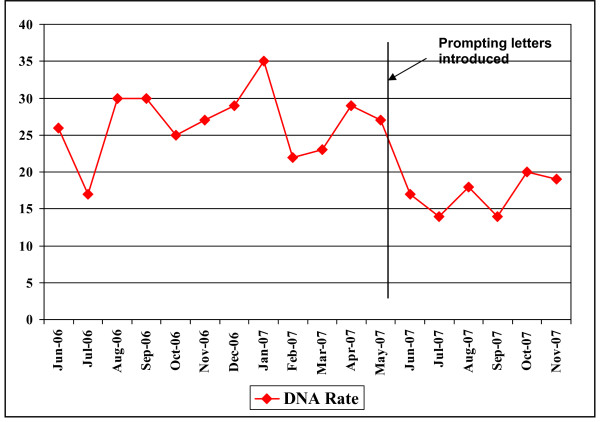
Non-attendance rates before and after prompt letters.

**Figure 2:**
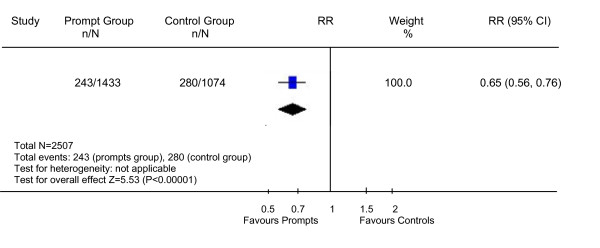
Relative risk of non-attendance in the prompt group compared to the control group.

A total of 1433 out-patient appointments were made at Bridge House from the months of June 2007 to November 2007. In total 1433 prompt letters were sent out in order to reach patients the week before their scheduled appointment. Figure 1 shows the number and percentage of non-attendances in this period. We compared these figures with the figures from June 2006 to November 2006 when no prompt letters were sent. The average non-attendance rate during June 2006 to Nov 2006 was 26% and this had dropped to 17% for the time period June 2007 to November 2007 following the introduction of prompt letters. The reduction in the non-attendance rate was statistically significant (RR 0.65, 95% CI 0.56 to 0.76, NNT 11) and is depicted in Figure 2. The average non-attendance rate during the same year from January 2007 to May 2007 was 27% (Figure 1). The non-attendance rates across the city in a similar community mental health team setting in the same period (June to November) had gone up from 22% in 2006 to 23% in 2007 and was not statistically significant (P = 0.45).

## Discussion

None of the patients seen in these outpatient clinics were what would be traditionally called 'new patients' as they had already been seen by the team and in effect they were follow up appointments. The prompting letters were only sent to patients who were booked into this model of 'outpatient clinics' and not to the ones who received an initial 'gate keeping appointment'. Therefore all patients in our sample had already engaged with secondary care mental health service which makes our sample different from that of previous studies. It is also likely that this model of care is not uniform across NHS Trusts. Previous studies have shown that prompts and information leaflets are helpful in reducing non-attendance for new rather than follow-up patients [15], however our study results suggest that prompt letters may be useful for improving attendance of follow-up patients.

The combined outpatient case load for both teams is about 575 patients. For the period of June 2006 to November 2006, 1074 appointments were made in the outpatient clinics and 1433 appointments for the corresponding period in 2007. Of these 1433 appointments, 1277 (89%) were for the same patients who were seen in 2006 which makes the two samples comparable with two caveats. Firstly, clinicians in 2007 might have acted differently towards patients compared to clinicians in 2006. These kinds of changes are unfortunately part of 'reality'. Secondly, there is a possibility that the reduction in non-attendance rates could have been due to the improvement in mental health and functioning of the patient population who remained largely constant. Non-attendance rate for patients new to 2007 (i.e. follow up patients seen only in 2007 and not in 2006) was 14.7% compared to 17.3% for patients who had remained constant between 2006 and 2007. This difference was not statistically significant (RR 1.17, 95% CI 0.79 to 1.74). Thus improvement in mental state of patients is less likely to have caused the reduction in non-attendance for 2007.

Researchers have been interested in finding out why patients miss their appointments rather than ring and cancel beforehand. There is some data to suggest that a third of patients forget or do not consider cancelling as they may be too embarrassed to call on the day and admit this [20]. In our study, if the patient or their carer informed us of their inability to attend, then their appointment was deemed as 'cancelled' and not as 'DNA'. The E-care system we used does not record cancellations and hence we were unable to clarify if some of the 'cancelled' appointments still showed up as DNAs thus underestimating the impact of our intervention. We are hoping that a new software 'Paris' which replaces 'E-care' will solve this problem for similar analysis in future. Cancellations were perceived to be on the rise by clinicians leading us to hypothesise that perhaps receiving a prompt letter made patient's feel more confident to ring and cancel even though it was close to their appointment date. Cancellations enabled us to use available resources more efficiently. Also to reiterate the importance of attending appointments, we have begun to display up to date monthly non-attendance figures in our outpatient waiting areas.

With each outpatient appointment costing about £70 to £80 in Leeds, this intervention was associated with an estimated savings of about £480 per week. Secondary benefits are yet to be evaluated. Reduction seen in the non-attendance rates could be related to several factors, however majority of our patient population remained constant and there was no major change in the way service was delivered. We compared the non-attendance rates from earlier in the year (January to May 2007) and found that there was no particular trend towards a decline in non-attendance rates prior to this intervention. We also compared the non-attendance rates in other parts of our service where prompting letters had not been introduced which showed that there was no change in their respective non-attendance rates between 2006 and 2007. As in any before and after study design, it is difficult to attribute change observed to the intervention alone due to possibility of various confounding variables or biases, however this intervention has already been evaluated in a pragmatic randomised controlled trial and findings pooled with an existing systematic review to prove its effectiveness [16]. One of the criticisms of randomised trials in the past has been that sample populations are narrowly defined and results are often difficult to generalise [21]. We were able to apply finding from this pragmatic trial [16] without much difficulty in a busy clinical setting and found results similar to conclusions arrived at within a trial setting. Perhaps this demonstrates that well designed pragmatic randomised controlled trials are able to produce results that are clinically relevant and repeatable in clinical settings. These results may only be applicable in similar settings within the UK or elsewhere that operates a similar outpatient system.

## Conclusion

Prompt letters have previously demonstrated to be effective in reducing non-attendance rates in RCT settings and this has been confirmed by systematic reviews as well. Our study was able to demonstrate that it could be possible to reduce non-attendance rates in a day to day clinical setting. Majority of the patient population remained constant during the study periods compared although there were changes in medical personnel. This makes it difficult to attribute change solely to the intervention alone. However this study was able to demonstrate that the results of pragmatic randomised trials can be easily applied to day to day clinical practice and it is possible to produce similar results in non-randomised settings. We found that prompting letters are a useful and easy to apply evidence based intervention to reduce non-attendance rates with a potential to achieve significant cost savings in similar mental health settings.

## Abbreviations

DNA stand for 'Did not attend' and NHS stands for 'National Health Service'.

## Competing interests

All authors are employed by the Leeds Partnerships NHS Foundation Trust. IK was part of the PROMPTS trial.

## Authors' contributions

MJ participated in the designing of the study, acquisition of data followed by analysis and interpretation of the data and the writing of the manuscript. RR participated in acquisition, analysis and interpretation of the data including the drafting and revisions of the manuscript. IK participated in the designing of the study and acquisition of data. All authors read and approved the final manuscript.

## Appendix 1

Example of letter

Organisation's Logo

PRIVATE & CONFIDENTIAL

***CMHT 10 & 11***,

***Bridge House, Balm Road***,

*Leeds LS10 2TP*

*Tel: 0113 xxxxxxx*

[Patient's name & address]

[Date]

Dear [Patient's name].

Re: Your appointment at Bridge House

This is a short reminder of your appointment at **Bridge House **on the **[Date at Time]**. Your appointment will be with **Dr. XX **and will last for xx minutes. This interview will be private and confidential. It is often helpful if you bring a friend or family member and medications along. Our clinic has a reception and once the receptionist knows you have arrived, she will inform the doctor.

Bridge House is located on Balm Road and a map with directions is enclosed with this letter.

If you have forgotten about the appointment or made other plans, do not worry. Please let me know at the above telephone number and we will rearrange your appointment at a time which is convenient for you.

[Name of Secretary]

Secretary to Dr XX

Encl. [Map]

## Pre-publication history

The pre-publication history for this paper can be accessed here:


